# Pneumatized Inferior Turbinate: A Report of Three Cases

**DOI:** 10.7759/cureus.29252

**Published:** 2022-09-17

**Authors:** Khalid Aldilaijan, Mai Almasoud, Ahmed El Baramawy, Jin-Young Min, Sung Wan Kim

**Affiliations:** 1 Otorhinolaryngology - Head & Neck Surgery, King Fahd Military Medical Complex, Dhahran, SAU; 2 Otorhinolaryngology - Head & Neck Surgery, NMC (New Medical Center) Royal Hospital, Abu Dhabi, ARE; 3 Otolaryngology, Kyung Hee University School of Medicine, Kyung Hee University Medical Center, Seoul, KOR

**Keywords:** pneumatized inferior turbinate, turbinate enlargement, turbinate pneumatization, inferior turbinate, inferior turbinate pneumatization, inferior concha bullosa

## Abstract

Inferior turbinate pneumatization is a rare cause of inferior turbinate enlargement. Suspected cases need careful examination and evaluation using computed tomography (CT) scan of paranasal sinuses followed by a tailored management plan. Here, we are reporting three cases of inferior turbinate pneumatization including their clinical assessment and CT scan of paranasal sinuses along with their surgical management. The case series is followed by a literature review of this entity and its management.

## Introduction

Unlike middle turbinate pneumatization, inferior turbinate pneumatization is a rare finding [1-4]. Patients with inferior turbinate enlargement can be asymptomatic or symptomatic. Symptomatic enlargement of the inferior turbinate mandates careful examination before and after local decongestant use. Further radiological evaluation may be needed before proceeding to definitive management.

Here, we are reporting three cases of inferior turbinate enlargement due to pneumatization of the inferior turbinate.

## Case presentation

Case 1

A 37-year-old lady presented to the ENT clinic complaining of persistent nasal obstruction for years, hyposmia, itching, sneezing, and on/off nasal discharge. No history of headache, trauma, or previous nasal surgery was noted. Nasal endoscopy showed bilateral inferior turbinate hypertrophy, bilateral nasal polyps confined to the middle meatus (grade I polyps), and moderately deviated nasal septum to the right side.

**Figure 1:**
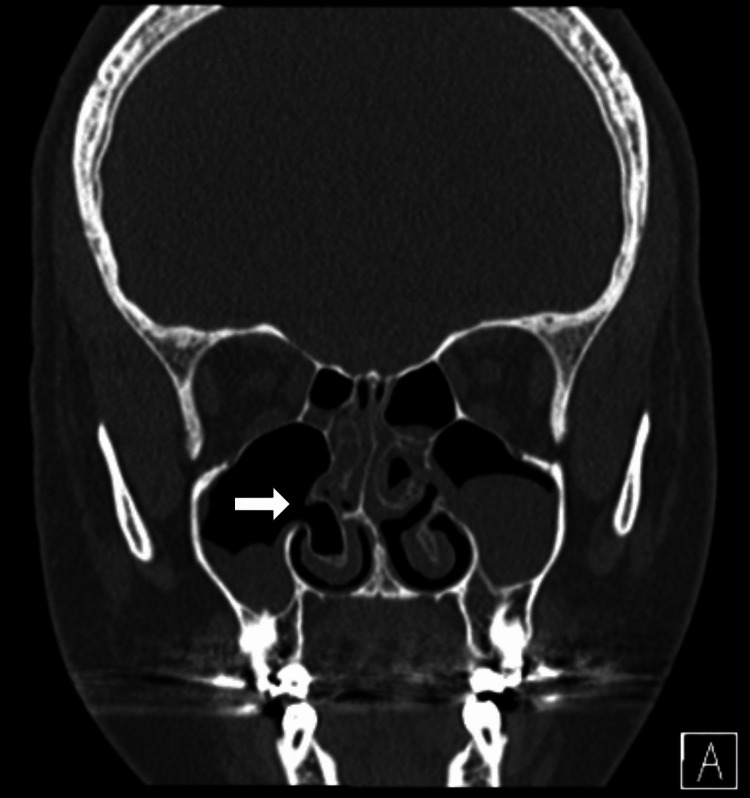
Non-contrasted paranasal sinuses CT scan, coronal view for case 1. Nasal findings include a pneumatized right-side inferior turbinate with a large opening into the ipsilateral maxillary sinus (arrow). Note that the inferior turbinate pneumatization pattern is communicating and show a mix between lamellar and bulbous types.

The patient showed no clinical improvement on nasal steroid (mometasone furoate) spray and saline irrigation. Computed tomography (CT) scan of paranasal sinuses (PNS) was done (Figure 1). Along with other sino-nasal findings, there was a pneumatized right-side inferior turbinate. The inferior turbinate pneumatization pattern was communicating with the maxillary sinus and showed a mix of lamellar and bulbous types.

She underwent endoscopic septoplasty, bilateral partial inferior turbinectomy, and bilateral functional endoscopic sinus surgery (FESS). During the procedure, the pneumatized portion of the right inferior turbinate was addressed by removal of the bony and mucosal components using a microdebrider resulting in a mega antrostomy. Hemostasis was achieved by minimal diathermy and by using an absorbable gelatin paste hemostatic matrix. A postoperative follow-up visit showed a patent nasal airway bilaterally with no synechia.

Case 2

A 14-year-old girl presented with bilateral nasal obstruction, hyposmia, and post-nasal drip (PND) along with recurrent facial pain and heaviness. Her mother had a history of chronic rhinosinusitis with nasal polyp (CRSwNP).

**Figure 2:**
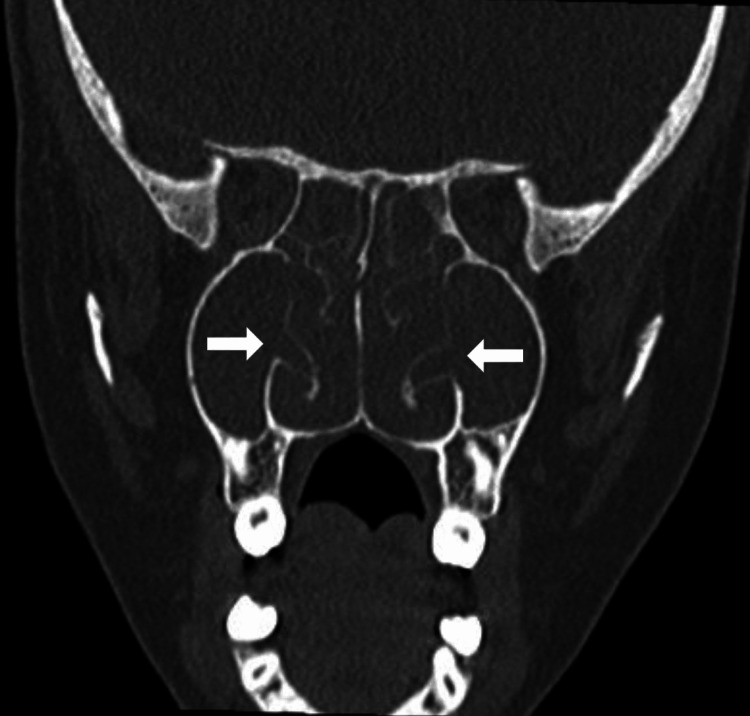
Non-contrasted paranasal sinuses CT scan, coronal view for case 2. There is a complete opacification of sino-nasal cavities on both sides due to nasal polyps with opacified lamellar pneumatization of the inferior turbinate bilaterally. The pneumatization is communicating with the maxillary sinus bilaterally (arrows).

Nasal endoscopy showed bilateral grade 4 nasal polyps. CT scan of PNS showed complete opacification of sino-nasal cavities on both sides with opacified lamellar pneumatization of the inferior turbinate bilaterally (Figure 2). Cystic fibrosis transmembrane conductance regulator (CFTR) gene screening was negative.

A minimal improvement was noticed both subjectively and objectively after using oral steroids, oral macrolides, and steroid nasal irrigation. The patient underwent image-guided FESS with bilateral partial inferior turbinectomy (while saving the anterior portion of inferior turbinates).

Case 3

A 24-year-old lady presented with bilateral nasal obstruction and rhinorrhea for two to three years. Physical examination revealed nasal septal deviation with bilateral inferior turbinate enlargement.

**Figure 3:**
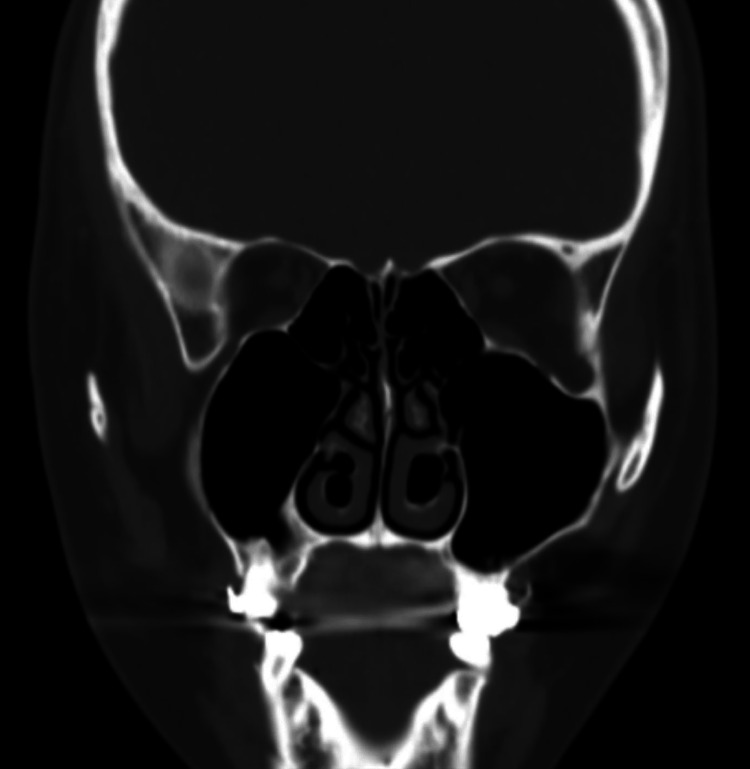
Non-contrasted paranasal sinuses CT scan, coronal view for case 3. It shows bilateral inferior turbinate pneumatization of the bulbous type with communication to the inferior meatus.

She did not improve on nasal steroids and oral antihistamines. Acoustic rhinometry showed no significant difference before and after nasal decongestant application. CT scan of PNS showed bilateral inferior turbinate pneumatization of bulbous type with communication to inferior meatus (Figure 3).

Septoplasty was done with bilateral inferior turbinoplasty by resecting the lateral lamella and then lateralization of the medial portion.

## Discussion

Concha bullosa is defined as middle turbinate pneumatization and can be of the superior or inferior turbinate whenever specified [1,5]. Inferior turbinate pneumatization is a rare finding [1-4]. Zinreich et al. first described it in 1988 [6]. Then over the next 20 years (until 2008), only 28 cases were described in the English literature [3].

The prevalence of inferior turbinate pneumatization varies between studies. This variation could be related to the difference in the study groups’ characters. In a group where a CT scan of sinuses was performed in symptomatic adults, Baldea et al. found that the prevalence of inferior turbinate pneumatization was 4.88% [4]. On the other hand and in CT scan sinuses that were not done specifically to address nasal complaints, Yang et al. found that the prevalence was 0.03% [3,7].

The large difference in pneumatization rate between inferior turbinate and middle turbinate is attributed to the different embryological origins of these conchal bones. The inferior turbinate originates from the maxilloturbinal prominence, while the middle turbinate originates from the first ethmoturbinal prominence [8].

In general, inferior turbinate enlargement can be asymptomatic or symptomatic. Symptoms of inferior turbinate pneumatization may include nasal obstruction, epiphora if it causes nasolacrimal duct obstruction, and facial pain in case of contact points [9-11]. Allergy testing like skin prick test could be useful in the assessment of patients with enlarged inferior turbinates and allergic rhinitis.

The ideal nasal examination includes nasal endoscopy before and after using a local decongestant. Differential diagnosis of inferior turbinate enlargement that does not respond to local decongestant should include on top of the list conchal bone structural abnormality like compact lamellar bone or enlargement due to inferior turbinate pneumatization. And yet, clinical examination may not always lead us to the right answers.

The technological advances in the field of radiology have allowed otolaryngologists to obtain a more objective assessment of nasal airway obstruction. So, a CT scan of PNS may be of good value for adults with suggestive clinical pictures [12]. Some authors have included CT scans of PNS as a useful tool for preoperative planning in patients with inferior turbinate enlargement [13].

The main aim of the CT scan here is to evaluate the type of inferior turbinate enlargement whether its type is thin lamella with enlarged soft tissue component, compact bulky lamella, or combined (where there is a compact lamella and enlarged soft component) [13].

The patterns of inferior turbinate pneumatization can be bulbous, lamellar, or extensive. Each can be either communicating or non-communicating with the maxillary sinus [13]. Once the cause and type of persistent inferior turbinate enlargement are identified, a surgical method can be chosen accordingly.

Asymptomatic inferior turbinate pneumatization can be left without intervention. On the other hand, those causing symptoms may need medical surgical management [14]. In a recent report, Alkhaldi et al. described two patients who had satisfactory symptom improvement on medical treatment. Our reported cases in this article were still symptomatic despite medical treatment [15]. For this reason, we discussed with the patients the need for surgical management.

The method of surgery can be tailored according to CT scan findings. The described surgical methods in the literature are many and include out-fracture and radiofrequency, resection of lateral lamella in the inferior turbinate, and resection of the pneumatized part using turbinectomy scissors [7].

The pattern of inferior turbinate pneumatization and communication was different among our reported patients. That is why we used different surgical approaches in them.

## Conclusions

Clinical and surgical approaches differ according to the cause of inferior turbinate enlargement. Differential diagnosis of enlarged inferior turbinate that shows no response to local decongestant may include inferior turbinate pneumatization. CT scan of sinuses is of good value in these cases. Rapid radiological advances and expansion in CT scan use lead to more discovered cases. Management of inferior turbinate pneumatization depends on its pneumatization pattern, communication area, and the presence of symptoms.
